# Management of patients with proximal femur fractures under DOACs

**DOI:** 10.1007/s00068-024-02472-4

**Published:** 2024-02-24

**Authors:** Marc Maegele

**Affiliations:** 1 Department for Trauma and Orthopedic Surgery, Cologne-Merheim Medical Center (CMMC), Witten/Herdecke University, Campus Cologne-Merheim, Ostmerheimer Str. 200, 51109 Cologne, Germany; 2 Institute for Research in Operative Medicine (IFOM), Witten/Herdecke University, Campus Cologne-Merheim, Ostmerheimer Str. 200, 51109 Cologne, Germany

**Keywords:** Proximal femur fractures, Direct oral anticoagulants (DOACs), Perioperative management, Bleeding, Reversal, Antidotes

## Abstract

**Purpose:**

In the past, preinjury direct oral anticoagulant (DOAC) intake has led to delays in time to surgery (TTS) in patients with proximal femur fractures and delays in surgery have been associated with impaired outcomes. Although healthcare institutions/federal committees have set rules for treatment within 24 h of injury, comprehensive guidelines for the perioperative management of these patients, in particular when on preinjury DOACs, are still lacking. This contribution aims to summarize the current evidence on the safe time window for surgery in patients with proximal femur fractures on preinjury DOACs and to outline therapeutic options if emergency DOAC reversal becomes necessary.

**Methods:**

Narrative review based upon selective review of the pertinent literature.

**Results:**

For the majority of patients with proximal femur fractures and on preinjury DOACs, early surgery appears safe as soon as medical clearance has been obtained. There may be an increase in the need for blood products but with data not yet conclusive. Work-up including assessment of remaining anticoagulant activity and potential reversal should be restricted to patients at risk for bleeding complications, in particular in the presence of renal/hepatic impairment. Methodology for rapid assessment of DOACs including quantitative/qualitative concentration levels is work in progress. In the case of bleeding, rapidly acting reversal agents are available.

**Conclusion:**

Preinjury DOAC use should not routinely delay surgery in patients with proximal femur fractures.

## Introduction

Proximal femur fractures are common in the elderly population due to decreased bone mineral density, mostly resulting from high rates of osteoporosis and frequent low falls as primary mechanism of injury [1]. Incidence rates show a wide range across countries, including sex differences. For example, the annual incidence of proximal femur fractures in the United States is 957/100,000 for women and 414/100,000 for men [2, 3]. In a German retrospective single-center analysis based upon ICD-10 codes, the number of surgically treated proximal femur fractures increased by 100% between 2016 and 2022 with the largest increase of 60% between 2020 and 2022 [4]. With demographic change, the rate of proximal femur fractures is even to rise further in patients > 50 years with 300,000 per annum in 1990 to an estimated 512,000 per annum by 2040 in the United States [5]. Worldwide, around six million cases are assumed each year by 2050 [6].

The demographic change does not only lead to an increasing incidence of proximal femur fractures, but also rises economic pressure on healthcare systems. Proximal femur fractures are usually associated with significant (co)morbidity, mortality, and costs. The average treatment cost for acute inpatient care was calculated up to 8050 Euros in a prospective observational cohort of 402 patients [7]; the largest share was related to inpatient care on intensive care and regular wards with 5360 Euros [7]. In the United States, the total cost of proximal femur fractures is expected to increase up to 16 billion $ within the upcoming two decades [5]. After 1 year of injury, only 40 to 60% of patients have regained their previous level of mobility and ability to perform activities of their daily living [8]. In result, proximal femur fractures are considered the most expensive osteoporosis-associated fractures in general.

## Treatment of proximal femur fractures

Unless there is significant comorbidity, the treatment of proximal femur fractures is surgery. Early surgical intervention within 48 h of injury was frequently associated with improved outcomes [9, 10], and within 24 h with less pain and length of stay in-hospital [11]. Vice-versa, postponing surgery > 48 h of injury has been shown to substantially increase both short- and long-term morbidity and mortality [9–11]. Based upon these findings, healthcare institutions and federal committees meanwhile prescribe treatment of proximal femur fractures within 24 h of injury.

The surgical strategies and principles remain early anatomical reduction and fixation as best options to reduce the risk of postoperative complications, e.g. non-union and avascular necrosis in fractured neck femurs [12]. In the advanced age group, early prosthetic replacement reduces morbidity and mortality associated with femur neck fractures and with the sliding hip screw (DHS) being among the available options for stable intertrochanteric fractures [12]. Intramedullary nailing is beneficial in treating intertrochanteric fractures with comminution and loss of lateral buttress and has also been proven to have an increased success rate in subtrochanteric fractures [12]. Depending on fracture type and mode of surgery, the amount of soft tissue trauma is variable.

## Direct oral anticoagulants (DOACs) in patients with proximal femur fractures

Comorbidities and polypharmacy are high among patients with proximal femur fractures and German data indicate that around half of all burden occurs in people with disabilities and need for care living either at home or in an institution [13]. Preinjury anticoagulant intake is frequent in elderly patients with proximal femur fractures, mostly due to underlying cardiovascular comorbidity [14]. Since their first approval in 2010, direct oral anticoagulants (DOACs) have quickly emerged into an attractive alternative to the long‐standing standard of care in anticoagulation, vitamin K antagonists (VKA), with more effective, safe, and convenient treatment options [15].

DOACs can be categorized into two main classes: (a) oral direct factor Xa inhibitors (i.e., rivaroxaban, apixaban, edoxaban, and betrixaban) and (b) direct thrombin inhibitors (i.e., dabigatran). The advantages of DOACs as compared to VKAs include fewer monitoring requirements, less frequent follow‐up, more immediate drug onset and offset effects, and fewer drug/food interactions [16]. As a result, DOAC prescriptions exceeded those for VKAs by 2013, with apixaban being the most frequently prescribed DOAC for patients with non-valvular atrial fibrillation (NVAF; [17]). In 2015, DOACs made up 56% of all anticoagulant prescriptions and are now the choice for many patients with AF or a history of stroke and/or transient ischemic attack (TIA; [18]). The half-lives of DOACs range between 5 and 27 h, depending on renal clearance, which may substantially prolong effective half-lives [19–21]. The main pharmacological properties of the DOACs yet approved and clinically used are summarized in Table 1. A recent literature survey showed that DOAC use in proximal femur fractures ranges between 1 and almost 20% but—as the proportion of patients is consistently on the rise—the reported overall DOAC use percentages are most likely underestimated and do not fully reflect actual rates [22, 23].

**Table 1:** Pharmacokinetics and pharmacodynamics of DOACs [23]

	Dabigatran	Rivaroxaban	Apixaban	Edoxaban	Betrixaban
Target	Thrombin (IIa)	Factor Xa	Factor Xa	Factor Xa	Factor Xa
T1/2 (h)	12–17	Young, healthy: 5–9 Elderly: 11–13	8–15	10–14	19–27
Tmax (h)	2	2–4	1–3	1–2	3–4
Bioavailability (%)	7	66	50	62	34
Renal excretion (%)	80	66	25	35	17.8
Fecal excretion (%)	82–88	26.4	46.7–56	62.2	85
CYP450 metabolism	No	Yes	Yes	No	No
Specific reversal agents	Idarucizumab	Andexanet alfa	Andexanet alfa	–	–

## Delay in time to surgery in patients on DOACs

Several studies have documented significant delays in time to surgery (TTS) among patients with proximal femur fractures on preinjury DOACs versus non-anticoagulated patients [23]. In a recent systematic review involving 34 studies with 39,449 patients, TTS was 13.7 h longer (95% confidence interval (CI) 9.8 to 17.5; *p* < 0.001) among patients on DOACs compared to those not on DOACs which translated into a threefold increased odds of surgery > 48 h from admission (odds ratio (OR) 3.0 (95% CI 2.1 to 4.3); *p* = 0.001; [24]). In most studies, though confounded by small sample size and retrospective in nature, the proportion of patients on DOACs that underwent surgery > 48 h was greater as compared to non-anticoagulated patients with proportions ranging between 9 and 49% and TTS between 28 and 67 h [23]. Potential reasons for these delays including their variation may include still existing uncertainties with these drugs in the perioperative management, waiting for DOAC activity to decrease in the absence of reversal, limited access to reversal agents, lack of clear guidelines for their management, as well as local-specific procedural differences [23]. However, increased TTS in patients on DOACs was not uniformly associated with increased mortality [23]. To date, the vast majority of studies found no difference in mortality rates between DOACs, VKA, and non-anticoagulated patients although patients on DOACs were largely operated > 48 h after admission [23–26].

## Monitoring of residual DOAC activity/levels

It is likely that a substantial proportion of patients has acceptable DOAC serum levels at the time of surgery, but another proportion may still have therapeutically active levels that potentially cause (excessive) bleeding [27–29]. In this context, the type, invasiveness of the surgical procedure, and the expected blood loss during the intervention become relevant. In a retrospective single-center experience on 308 surgical interventions in 298 patients, only in surgeries with a high expected blood loss (> 500 ml) a calculated rivaroxaban concentration > 100 mg/l was associated with a significant increase of perioperative blood loss [30]. The blood loss increased with rising rivaroxaban concentrations from 575 ml at concentrations of 20 mg/l or less up to 1400 ml at concentrations > 100 mg/l [30]. Recent studies have indicated that emergency patients under DOAC treatment show a high variation of anticoagulant concentrations at baseline and that the physiological clearance of DOACs is not as predictable as initially thought with renal insufficiency/chronic kidney disease (CKD) substantially prolonging clearance per se [31]. DOAC serum cut-off levels for safe early surgery ranging between 30 and 80 ng/ml have been proposed but not yet been substantiated by any evidence nor by broader consensus [32, 33]. Furthermore, there may be interindividual variation in achieved drug levels, intraindividual fluctuations in DOAC plasma concentrations, or lack of adherence to therapy in individual patients [34]. In cases with acceptable concentration levels, unnecessary administration of reversal procoagulants or even antidotes would directly increase the thromboembolic risk. The measurement of DOAC concentrations in these cases and prior to any kind of intervention could therefore provide useful additional information to enable further decision-making more sensibly and safely. At least since the availability of specific antidotes, there has been growing interest for quantitative laboratory diagnostics and point-of-care tests (POCTs), especially if there is uncertainty regarding type, dose, and timing of last DOAC intake.

Global coagulation tests, e.g. prothrombin time (PT)/international normalized ratio (INR) or activated partial thromboplastin time (aPTT), and thrombin time (TT) and anti-factor Xa activity (AXA) are variably influenced by the different DOACs as well as by the reagents used (Table 2; [35–37]). The quantitative assessment of dabigatran and of the three available factor Xa inhibitors apixaban, rivaroxaban, and edoxaban is possible through substance-specific calibrated tests which are recommended as “gold standard.” However, these measurements are not widely implemented and available 24/7 and if so, long turn-around times even in emergency settings and in larger centers should be anticipated [38, 39]. To date, there are no substance-specific calibrated POCTs yet available for rapid bedside use. DOAC urine dipsticks can be used to reliably exclude or detect urine concentrations below or above 95 ng/ml thereby differentiating dabigatran from factor Xa inhibitors within 10 min of test time [40]. However, they do not provide any information on current plasma concentrations and the extent of remaining anticoagulation [41]. Viscoelastic test assays may provide fast and essential point-of-care information when DOAC-specific assays are being used but these need further refinement [42]. The identification and quantification of residual DOAC plasma concentrations with DOAC non-specific viscoelastic assays is not sensitive enough compared to recommended and calibrated anti-Xa laboratory measurements [42].

**Table 2:** DOAC effects on laboratory assays and measurements. The accuracy of the diluted thrombin time (dTT) decreases with lower plasma concentrations of dabigatran. A non-diluted TT may confirm the absence of active dabigatran concentrations. However, the TT—depending on the method used—is so sensitive and maybe prolonged even in the presence of very low plasma concentrations although the risk of bleeding is not evident. A maximum two-fold prolonged TT appears still sufficient to exclude relevant plasma levels. A prolonged aPTT indicates relevant dabigatran concentrations but not vice-versa! Low assay-specific cut-offs to exclude dabigatran, rivaroxaban, and edoxaban concentrations > 30 and > 50 ng/ml have been suggested for the HC-POCT Hemochron Signature Elite (IL-Werfen; [44]). Prolonged PT/INR may indicate relevant anticoagulation for rivaroxaban and edoxaban but not vice-versa! In a time-sensitive emergency, low but relevant concentrations of both can be safely excluded if reagent-specific cut-offs for the respective global coagulation tests have been established (CoaguCheck (Roche Diagnostics) and HC-POCT Hemochron Signature). However, this is not valid for the assessment of apixaban concentrations! To quantify the different factor Xa inhibitors, substance-specific calibrated tests are the “gold standard.” Anti-factor Xa activity is affected by the intake of factor Xa inhibitors and absence of activity generally confirms absence of corresponding anticoagulant concentrations! The latter assessment can be used in the absence of substance-specific calibrated tests but with some limitations! If the last DOAC intake was within the interval in time-to-peak (2–4 h), there may be a risk that the anticoagulant activity is still developing and increasing concentrations may only be picked up through sequential assessment!

		Dabigatran	Rivaroxaban	Apixaban	Edoxaban
Routine diagnostics	Activated partial thromboplastin time (aPPT)	(↑)—↑	(↑)	(↑)	(↑)
	Prothrombin time PT/INR	(↑)	↑—↑↑	(↑)	↑—↑↑
Available, non-specific but sensitive tests	Thrombin time (TT)	↑↑↑	N/A	N/A	N/A
	Ecarin clotting time	↑↑↑	N/A	N/A	N/A
	Anti-factor Xa activity	N/A	↑↑↑	↑↑↑	↑↑↑
Specific and sensitive tests		Dabigatran-calibrated diluted TT or direct thrombin inhibitor assay	Rivaroxaban-calibrated anti-factor Xa activity	Apixaban-calibrated anti-factor Xa activity	Edoxaban-calibrated anti-factor Xa activity

## Safety of early surgery

There is increasing evidence that early surgery in patients with proximal femur fractures on preinjury DOACs is safe. Several studies have shown no increase in blood loss or transfusion rates in patients under preinjury DOAC medication in need of surgery for proximal femur fractures in general [23]. A systematic review and meta-analysis of data from 21,417 patients on hemostatic complications reported no differences between DOAC and non-anticoagulated patients in respect to estimated blood loss and the frequency of blood transfusion [43]. In a retrospective cohort of patients > 65 years treated either with closed reduction and internal fixation or with hemiarthroplasty < 48 h or > 48 h after admission, there was no difference in perioperative hemoglobin change, rate of transfusion, and hematoma formation in DOAC patients versus non-DOAC patients versus non-anticoagulated patients [45]. Similar results were reported from other studies [46, 47], even when adjusted for known differences in estimated blood loss between short and long cephalomedullary nailing [47]. In a recent systematic review and meta-analysis, five retrospective cohort studies included DOAC patients (*n* = 223) versus non-anticoagulated controls (*n* = 1750) undergoing expedited surgery within 48 h after admission [48]. Preinjury DOAC use was neither associated with increased length of surgery nor 30-day mortality but with a higher rate of perioperative blood transfusion (OR 0,58, 95% CI 0,36–0,96; [48]). The rate of DOAC reversal was low in these studies, with most protocols calling for no reversal before surgery within 24 h of admission. One study included only patients with sub- or intertrochanteric fractures which had been treated with cephalomedullary nail fixation rather than arthroplasty within 24 h of admission [49]. As this approach is known to be associated with higher surgical blood loss per se, any coexisting hemostatic impairment may be amplified under this condition. In the absence of preoperative DOAC reversal, the risk for perioperative blood transfusion in this study among DOAC users versus non-anticoagulated patients was increased by factor 3,4; however, transfusion here was mainly driven by significantly lower hemoglobin levels upon admission [49]. This result is similar to another retrospective cohort reporting slightly higher transfusion rates in DOAC patients versus non-users if operated < 24 h after admission (RR 1,14 [1.02–1.27]; [26]). However, neither hemoglobin nor hematocrit data were reported in the later study. These data may suggest an increased need for blood products in patients on preinjury DOAC medication and surgery < 24 h after admission but with the data yet available difficult to interpret. Overall, mortality in patients after surgery for proximal femur fractures and operated within 24 h after admission seems rather be driven by high ASA grade reflecting pre-existing comorbidities and overall patient state than by type of preinjury anticoagulation [50].

## DOAC reversal

Increased perioperative blood loss with subsequent use of blood products has not consistently been reported from patients with proximal femur fractures and preinjury DOAC medication, but some patients may bleed or start bleeding intraoperatively in the presence of residual DOAC activity. In these patients, pharmacological DOAC reversal appears to be an attractive option for bleeding control and to mitigate further concern for surgical bleeding. In the past, prothrombin complex concentrate (PCC) which contains highly concentrated coagulation factors (II, IX, and X in three-factor [3-F] PCC and II, VII, IX, and X in four-factor [4-F] PCC) has been used off-label with the aim to boost factor levels and, thereby, to “overwhelm” the inhibitors [51]. This means that PCC does not directly inhibit DOACs, nor does it affect FXa levels in general [52]. 4F-PCC also does not address adequately DOAC-induced inhibition of thrombin generation (TG); it is only able to normalize TG over a low and narrow range of FXa inhibitor concentrations, e.g. < 75 ng/ml [52]. In a prospective multicenter cohort study with patients on apixaban or rivaroxaban, reversal was effective in 68% with a fixed dose of PCC 2000 units [53].

Meanwhile, two specific agents have been approved for DOAC reversal: (a) idarucizumab for the reversal of dabigatran and (2) andexanet alfa for the reversal of apixaban and rivaroxaban (Table 3). Data on edoxaban is still non-sufficient for approval in most countries except Japan. Idarucizumab and andexanet alfa have been licensed by the European Medicines Agency (EMA) and the Food and Drug Administration (FDA) for emergency surgery/urgent procedures, and life-threatening or uncontrolled bleeding (idarucizumab) or for patients treated with apixaban or rivaroxaban, when reversal of anticoagulation is needed owing to life-threatening or uncontrolled bleeding (andexanet alfa). Idarucizumab is given in a 5-g dose at two 50-ml bolus infusions of 2.5 g each and no more than 15 min apart [51]. Andexanet alfa is given as a bolus followed by a 2-h infusion in a low-dose or high-dose regimen to avoid rebound FX activity [51]. The recommended dosing, i.e. low or high dose, depends on the specific FXa inhibitor, the dose of FXa inhibitor, and the time since last intake. Both agents reverse DOAC activity within minutes of administration without any dose adjustment, neither for elderly nor for renal and hepatic impairment [54]. A systematic review and meta-analysis assessed clinical outcomes with the use of 4-factor PCC (*n* = 2688), idarucizumab (*n* = 1111), or andexanet (*n* = 936) for reversal of severe DOAC-associated bleeding based upon data retrieved from 60 studies involving 4735 patients [55]. The effective hemostasis rate was 78.5% (95% CI: 75.1 to 81.8%) and was similar regardless of the reversal agent considered; the overall rate of thromboembolic events was 4.6% (95% CI: 3.3 to 6.0%), being higher for andexanet alfa (10.7%; 95% CI: 6.5 to 15.7%). The overall risk of death was markedly and significantly associated with failure to achieve effective hemostasis (relative risk: 3.63; 95% CI: 2.56–5.16; [55]).

**Table 3:** Characteristics of the two available DOAC specific antidotes

	Idarucizumab (Praxbind®)	Andexanet alfa (Ondexxya®)
Reversal agent for	Dabigatran	Factor Xa (FXa) inhibitor (apixaban, rivaroxaban)
Mechanism of action	Humanized monoclonal antibody fragment that binds dabigatran with high affinity and specificity	Recombinant inactive human FXa that binds and sequesters FXa inhibitor molecules thereby rapidly reducing anti-FXa activity
Trials	RE-VERSE AD	ANNEXA-A, -R and -I, ANNEXA-4
Dosing	5 g (2 vials of 2.5 g/50 ml) and a second dose if needed	*Low dose:* 400 mg (rate 30 mg/min) bolus and 4 mg/min for 120-min infusion *High dose:* 800 mg (rate 30 mg/min) bolus and 8 mg/min for 120-min infusion
Indications	Life-threatening or uncontrolled bleeding on dabigatran	Life-threatening or uncontrolled bleeding on apixaban or rivaroxaban
Safety	4.8% thromboembolic events within 30 days	10% thromboembolic events within 30 days

To date, the ANNEXA-I study is the first randomized head-to-head comparison between andexanet alfa (75% of study patients with low-dose andexanet alfa) and usual care (87% of study patients with PCC as part of standard care) in patients with acute hemorrhage under an oral factor Xa inhibitor. The study was conceptualized as a phase 4, multicenter, prospective, randomized, open-label, blinded-endpoint trial in patients with acute intracranial hemorrhage treated with FXa inhibitors [56]. The primary efficacy endpoint was composite effective hemostasis at 12 h and the study was terminated prematurely due to superiority of andexanet alfa at interim analysis with 95% reduction of anti-FXa activity from baseline to nadir at 2 h post-randomization versus 23% with standard care [57]. The adjusted absolute increase in effective hemostasis with andexanet alfa versus usual care in the primary efficacy population (*n* = 452) was 13.4% (*p* = 0.003) and 11% in the extended population (*n* = 530), respectively (*p* = 0.008; [57]). The rate of thromboembolic events was similar to the one observed in the referenced meta-analysis as well as in the registration studies at around 10% [55].

Current guideline recommendations for the rapid reversal of DOAC anticoagulation in the acute setting differ from each other. While there is broad consensus on the use of idarucizumab for the emergency reversal of dabigatran [58, 59], the recently updated European Society of Anaesthesiology and Intensive Care (ESAIC) guideline on the management of severe perioperative bleeding favors the use of PCC over andexanet alfa in bleeding patients treated with anti-FXa agents, e.g. rivaroxaban, apixaban, and edoxaban [58]. In contrast, the 2023 updated European guideline on the management of major bleeding and coagulopathy following trauma in its 6th version recommends reversal with andexanet alfa if bleeding is life-threatening in the presence of an apixaban or rivaroxaban effect [59]. If andexanet alfa is not available, or in patients receiving edoxaban, PCC may be considered (25–50 U/kg; [58]). The newly formed task force on the “Clinical guideline on reversal of direct oral anticoagulants in patients with life threatening bleeding” in their yet unpublished statement sees PCC and andexanet alfa as equal option for reversal in the perioperative setting including severe bleeding based upon the current evidence. Consensus recommendations also consider the use of antifibrinolytic tranexamic acid (TXA) as adjuvant in the acute setting but in the absence of clear evidence [60].

## Preoperative management

The question of prophylactic reversal prior to early surgery remains under debate and neither idarucizumab nor andexanet alfa have yet received approval for this indication. Very few observational studies of limited quality suggest that preoperative DOAC reversal may not be necessary. In a retrospective multicenter cohort of 459 geriatric patients, not reversing anticoagulants was not associated with increased blood loss or transfusion requirement when compared to reversal [61]. The upcoming randomized phase-3 ANNEXA-RS study will address this issue by comparing safety and efficacy of andexanet alfa versus usual care in terms of preventing major blood loss in patients on factor Xa inhibitor treatment who require urgent surgery or an invasive procedure [62]. The study will enroll about 800 patients and will be initiated shortly. Evidence-based protocols to standardize perioperative management of patients with proximal femur fractures on DOACs have been published for different settings but need validation and further refinement [63].

## Conclusion

For the majority of patients with proximal femur fractures and on preinjury DOACs, early surgery appears safe as soon as medical clearance has been obtained. Further work-up including assessment of remaining anticoagulant activity levels and potential reversal should be restricted to patients at risk for bleeding complications, in particular those with renal/hepatic impairment. Methodology for rapid assessment of DOACs including quantitative and qualitative concentration levels is work in progress. Multidisciplinary collaboration is key and all patients should be prepared for possible intraoperative transfusion of blood products. In the case of bleeding, rapidly acting reversal agents are available.

## Data Availability

No datasets were generated or analysed during the current study.
